# *Ephedra fragilis* plant extract: A groundbreaking corrosion inhibitor for mild steel in acidic environments – electrochemical, EDX, DFT, and Monte Carlo studies

**DOI:** 10.1515/biol-2022-1050

**Published:** 2025-03-26

**Authors:** Mohamed Adil Mahraz, Rajae Salim, El Hassania Loukili, Abdelouahid Laftouhi, Salima Haddou, Amal Elrherabi, Mohamed Bouhrim, Rashed N. Herqash, Abdelaaty A. Shahat, Bruno Eto, Belkheir Hammouti, Zakia Rais, Mustapha Taleb

**Affiliations:** Laboratory of Engineering, Electrochemistry, Modelling and Environment, Faculty of Sciences Dhar El Mahraz, Sidi Mohammed Ben Abdellah University, Fez, 30050, Morocco; Euromed University of Fes, UEMF, Fes, Morocco; Laboratory of Applied and Environmental Chemistry (LCAE), Mohammed First University, Faculty of Sciences, B. P. 717 60000, Oujda, Morocco; Laboratory of Bioresources, Biotechnology, Ethnopharmacology, and Health, Faculty of Sciences, Mohammed First University, Oujda, B.P. 717, Morocco; Biological Engineering Laboratory, Faculty of Sciences and Techniques, Sultan Moulay Slimane University, Beni Mellal, 23000, Morocco; Laboratoires TBC, Laboratory of Pharmacology, Pharmacokinetics, and Clinical Pharmacy, Faculty of Pharmaceutical and Biological Sciences, P.O. Box 83, F-59000, Lille, France; Department of Pharmacognosy, College of Pharmacy, King Saud University, Riyadh, 11451, Saudi Arabia; Laboratory of Advanced Materials and Process Engineering, Faculty of Science, University Ibn Tofail, University Street, B.P 242, Kenitra, Morocco

**Keywords:** *Ephedra fragilis*, corrosion inhibition, electrochemical techniques, bioactive compounds, SEM analysis

## Abstract

The present study introduces an innovative approach to sustainable corrosion inhibition by utilizing the aerial parts of *Ephedra fragilis* (EF) as a natural inhibitor for steel in hydrochloric acid solutions. Unlike conventional synthetic inhibitors, EF extracts offer an eco-friendly and renewable alternative, emphasizing their potential for industrial applications. Both water and ethanolic extracts were evaluated, and their bioactive compounds were identified using high-performance liquid chromatography. The ethanolic extract was rich in *p-coumaric acid*, *sinapic acid*, and *hydrated catechin*, while the aqueous extract predominantly contained *catechin*, *gallic acid*, and *3-hydroxybenzoic acid*. Electrochemical techniques, including open circuit potential, electrochemical impedance spectroscopy, and potentiodynamic polarization, demonstrated remarkable corrosion inhibition efficiency, reaching up to 97%. The extracts exhibited mixed-type inhibition behavior, with efficiency improving as the concentration increased. Specifically, inhibition efficiencies of 96.13 and 96.84% were achieved using the Tafel method, highlighting the superior performance of EF extracts compared to many synthetic counterparts. Furthermore, scanning electron microscopy revealed the formation of a dense, protective organic layer on the steel surface, which underpins the high inhibition efficiency. This study not only validates the use of EF as an efficient, sustainable corrosion inhibitor but also opens new avenues for the integration of plant-based inhibitors into industrial practices, providing a long-term, eco-friendly solution to steel corrosion challenges.

## 1 Introduction

Corrosion is a natural process involving the gradual deterioration of metallic materials, resulting in significant economic losses and safety concerns. This phenomenon can be triggered by both natural and artificial activities [1,2,3,4]. In industries relying heavily on mild steel (MS) infrastructure, corrosion rates are particularly severe in acidic environments, leading to annual financial losses estimated in billions of dollars. Therefore, preventing acid–metal interaction is a critical priority. Organic inhibitors, when used in low concentrations, form a protective barrier that mitigates corrosion by reducing the direct contact between metals and acids [5,6,7]. Depending on the extraction or synthesis methods employed, these inhibitors can be broadly classified into synthetic and green inhibitors. Synthetic inhibitors, which are often derived from petrochemical processes, tend to be effective but come with drawbacks such as high toxicity, limited biodegradability, and environmental hazards. On the other hand, green inhibitors, primarily derived from natural sources, such as plants, amino acids, and biopolymers, offer significant advantages, including biodegradability, low toxicity, and environmental sustainability. This distinction is crucial for developing corrosion protection strategies that balance industrial needs with ecological responsibility. Several studies have explored the potential of plant extracts as green corrosion inhibitors, emphasizing their effectiveness and environmental benefits. Some plant leaves possess natural properties that enable them to act as effective corrosion inhibitors, providing an eco-friendly alternative to traditional chemical products [8–11]. These findings underscore the versatility of plant-derived inhibitors in addressing corrosion challenges across different acidic environments.

The use of plant extracts as corrosion inhibitors presents an innovative and sustainable approach. Plant-based inhibitors are particularly noteworthy due to their rich composition of bioactive compounds, such as flavonoids, alkaloids, phenolic acids, and other phytochemicals, which exhibit high efficiency in forming protective layers on metal surfaces. These compounds not only inhibit corrosion but also offer additional advantages, such as antioxidant, antifungal, and antibacterial properties. Importantly, plant extracts are renewable, widely available, and environmentally friendly, making them a promising alternative to conventional synthetic inhibitors. Key characteristics of effective corrosion inhibitors include their biodegradability, ensuring their decomposition naturally without any long-term environmental harm, and their non-toxicity, making them safe for human use while avoiding ecological contamination [12,13,14,15]. Additionally, the availability of raw materials for inhibitor production and the emphasis on environmental sustainability are crucial factors in their selection, as they ensure a balance between industrial efficiency and ecological conservation [16,17]. Despite the variety of corrosion inhibitors available on the market, many are expensive and toxic, posing risks to human health and the environment [18,19]. This highlights the urgent need for environmentally friendly corrosion inhibitors that provide effective protection while being cost-effective and safe. In response to this challenge, research has increasingly focused on green corrosion inhibitors derived from natural sources such as amino acids, proteins, plant extracts, cellulose, and starch [20,21,22,23]. The effectiveness of plant-based corrosion inhibitors is influenced by factors such as (a) the chemical composition of the inhibitors, (b) the properties of the metal surface, and (c) the electrical charge of the metal. Plant-derived compounds with heterocyclic structures, such as steroids, alkaloids, and flavonoids, have shown significant corrosion inhibition properties [6,24–27]. Numerous studies have demonstrated the strong potential of plant-based inhibitors, particularly due to their ability to adhere to metal surfaces and form protective layers against corrosion processes. *Ephedra fragilis* (EF) [28,29,30], locally known in Morocco as alnda, is a gymnosperm species widely distributed across the western Mediterranean region, including parts of southern Europe, North Africa, Madeira, and the Canary Islands. This plant belongs to the family Ephedraceae and comprises approximately 70 recognized species with notable pharmacological properties. Studies conducted in Morocco have identified key phytochemical constituents of EF, including flavonoids, alkaloids, and phenolic acids, which exhibit various biological activities. Similar research in Tunisia has revealed the presence of ferulic acid, luteolin-7-*O*-glucoside, myricetin, and kaempferol 3-*O*-rutinoside in the plant's aerial parts [31,32,33,34].

These compounds are known for their antioxidant, antifungal, antibacterial, and anti-inflammatory properties [18,19,35,36]. However, to date, no research has specifically explored the corrosion-inhibiting potential of the aerial parts of EF. Investigating this aspect could unveil effective, environmentally friendly solutions for corrosion prevention. Such findings would not only reduce maintenance costs but also mitigate the environmental impact of metal corrosion. This study aims to highlight the novelty and importance of plant extracts as corrosion inhibitors, providing insights into their chemical composition and mechanisms of action, while addressing the growing demand for sustainable industrial solutions.

## 2 Materials and methods

### 2.1 Preparation of MS

This research concentrates on choosing suitable materials, with MS being the primary selection. MS is favored in numerous industries for its affordability and favorable conductivity. Although it may contain other elements, carbon plays a crucial role in defining the steel's characteristics. A comprehensive chemical composition of MS is given in Table 1.

**Table 1:** Mass fraction of the chemical composition of the steel components used

Elements	Fe	Si	C	Mn	S	P	Al
(%) Mass	99.21	0.38	0.21	0.05	0.05	0.09	0.01

### 2.2 Electrolytic medium

In this research, the corrosive solution of focus was hydrochloric acid (HCl), prepared to a specific molarity by diluting 37% HCl with distilled water. The final mixture had a density of 1.19. The inhibitors under investigation were formulated using 1 M HCl and varied in concentration from 0.25 to 1 g/L.

### 2.3 Sample collection and authentication

**Figure 1:**
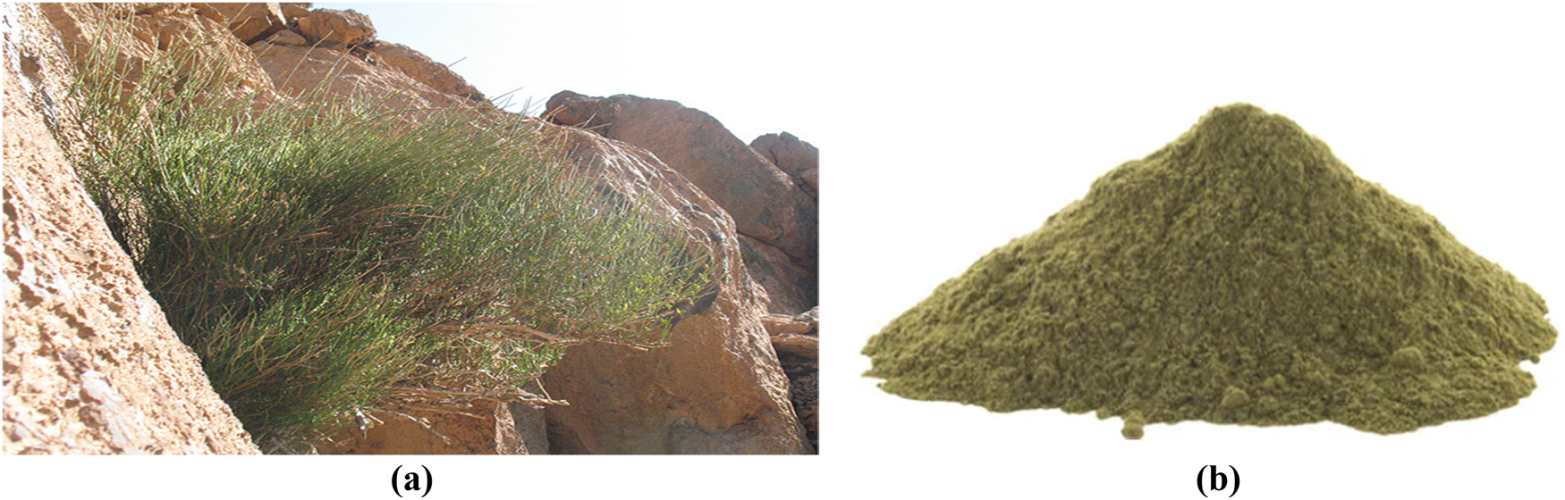
EF plant (a) and powder (b).

The plant Ephedra fragilis (EF) was collected based on ethnopharmacological grounds (Figure 1). This collection was conducted in partnership with local authorities and adhered strictly to the United Nations Convention on Biodiversity. A local healer was instrumental throughout this procedure. Professor Amina Bari, a botanist at the Faculty of Science, Dhar Mahraz, Fes, confirmed the plant's identity. Moreover, a reference specimen (320EF02 EF 008) was formally cataloged in the herbarium of the Botany Department.

### 2.4 Inhibitor preparation

The process of preparing EF extract as a corrosion inhibitor began with the collection of the plant’s aerial parts, which were then dried to remove any moisture. After drying, the aerial parts were ground into a fine powder using a mortar and pestle. This powder was then used for extracting the active compounds. For aqueous extraction, 100 g of EF powder was mixed with 350 mL of water in an appropriate container, and the mixture was stirred continuously for 72 h to ensure thorough extraction of the active compounds. After this period, the mixture was filtered through Whatman No. 2 filter paper (125 mm) to separate the aqueous extract from the solid plant material. The filtered extract was then concentrated using a rotary evaporator under reduced pressure at temperatures above 90°C, allowing the water to evaporate while preserving the active compounds. Once the solvent evaporated, the extract was dried in an oven at a constant temperature to remove any remaining moisture, yielding a dry extract that was stored at 4°C for later use. In parallel, an ethanol extraction was performed using the Soxhlet method. About 150 g of EF powder was subjected to continuous extraction with 300 mL of ethanol for 6 h. This method efficiently extracted the lipophilic and hydrophobic compounds from the plant. After extraction, ethanol was evaporated under vacuum at temperatures below 40°C to prevent thermal degradation of the active compounds. The resulting concentrated residue was also stored at 4°C for future use [37–39]. Through these aqueous and ethanol extraction processes, the active compounds from EF were isolated and prepared for use as corrosion inhibitors in various environments.

### 2.5 High-performance liquid chromatography with diode array detection (HPLC-DAD)

Extracts from the aerial part of the EF plant, encompassing both the ethanolic and aqueous extracts, were made at a concentration of 50 mg/mL. Subsequently, these extracts underwent filtration through 0.45 μm microfilters. Phenolic compounds were analyzed using HPLC equipped with a UV detector operating between 210 and 400 nm. A 40 µL sample was injected into a reverse-phase C18 column (250 mm × 4 mm, 5 μm), employing the following elution gradient: 20% B for 0–25 min, 100% B for 25–30 min, and returning to 20% B for 30–35 min. The mobile phases used were A (water with 0.5% phosphoric acid) and B (methanol), with a flow rate of 1 mL/min. The separation process was maintained at a constant temperature of 40°C. Identification of compounds was conducted according to established methods from previous research [40–43].

### 2.6 Electrochemical technique

Electrochemical assessments were conducted using a VersaSTAT 4 potentiostat, managed through VersaStudio software. The configuration comprised a small glass cell featuring three electrodes: the working electrode, which was a 1 cm^2^ MS sample; the reference electrode, which was an Ag/AgCl electrode; and the counter electrode, made from platinum wire. The MS sample, cut into a rectangular form with a 1 cm^2^ surface area, was immersed in the sample solution for 30 min to allow the establishment of an open circuit potential (OCP) [44–46].

### 2.7 Surface analysis

Scanning electron microscopy (SEM)–energy-dispersive X-ray (EDX) spectroscopy was used to examine the surface structure and composition of MS samples under two conditions: one without any inhibitor and another with an inhibitor present at a concentration of 1 g/L [40,47,48]. The analysis was conducted using an environmental scanning electron microscope (QUANTA 200 model), which was equipped with an EDX probe operating at an accelerating voltage of 15 kV. This technique allowed for detailed observation of the elemental composition and surface morphology of the MS, revealing any modifications caused due to the inhibitor’s presence.

### 2.8 Quantum chemical calculations

The hypothetical analysis presents a fascinating and valuable approach to understand corrosion inhibition by linking the reactivity of molecular structures with their inhibition efficacy. To explore this, density functional theory (DFT) calculations were carried out using the B3LYP/6-311G(d,p) method with Gaussian09 and GaussView 5.0.8 software. These calculations were performed in an aqueous environment to accurately replicate experimental conditions. The study investigated several global descriptors, including *E*_HOMO_, *E*_LUMO_, energy gap (Δ*E*_gap_), global hardness (*η*), global softness (*σ*), electronegativity (*χ*), and the fraction of electrons transferred (ΔN110) [49]. They were obtained using the following equations, where ΦFe/110 is 4.82 eV and *η*Fe (110) = 0 eV:

(1) \[\Delta{E}_{\text{gap}}={E}_{\text{LUMO}}-{E}_{\text{HOMO}},]\]

(2) \[\chi =\frac{1}{2}({E}_{\text{HOMO}}+{E}_{\text{LUMO}}),]\]

(3) \[\eta =\frac{1}{2}({E}_{\text{LUMO}}-{E}_{\text{HOMO}}),]\]

(4) \[\sigma =\frac{1}{\eta },]\]

(5) \[\Delta {\varepsilon }_{\text{back}-\text{donation}}=\frac{-\eta }{4},]\]

(6) \[\Delta{N}_{\text{metal}}=\frac{{\Phi}_{\text{metal}}-{\chi }_{\text{inh}}}{2({\eta }_{\text{metal}}+{\eta }_{\text{inh}})}=\frac{{\Phi}_{\text{metal}}-{\chi }_{\text{inh}}}{2{\eta }_{\text{inh}}}.]\]

Monte Carlo simulations were used to explore the adsorption characteristics of the tested molecules and their interactions with the iron surface. The simulated system consisted of inhibitor molecules, 180 water molecules, an acidic solution with 6 H_3_O⁺ ions and 6 Cl^−^ ions, and an iron steel surface. Before running the simulations, all components were optimized using the COMPASS force field. The simulations were performed with the Adsorption Locator module in Materials Studio 7.0. To ensure sufficient depth, the Fe (1 1 0) crystal was initially created with a 30 Å edge and then expanded into a supercell measuring 10 × 10 [50].

## 3 Results and discussion

### 3.1 HPLC analysis

**Figure 2:**
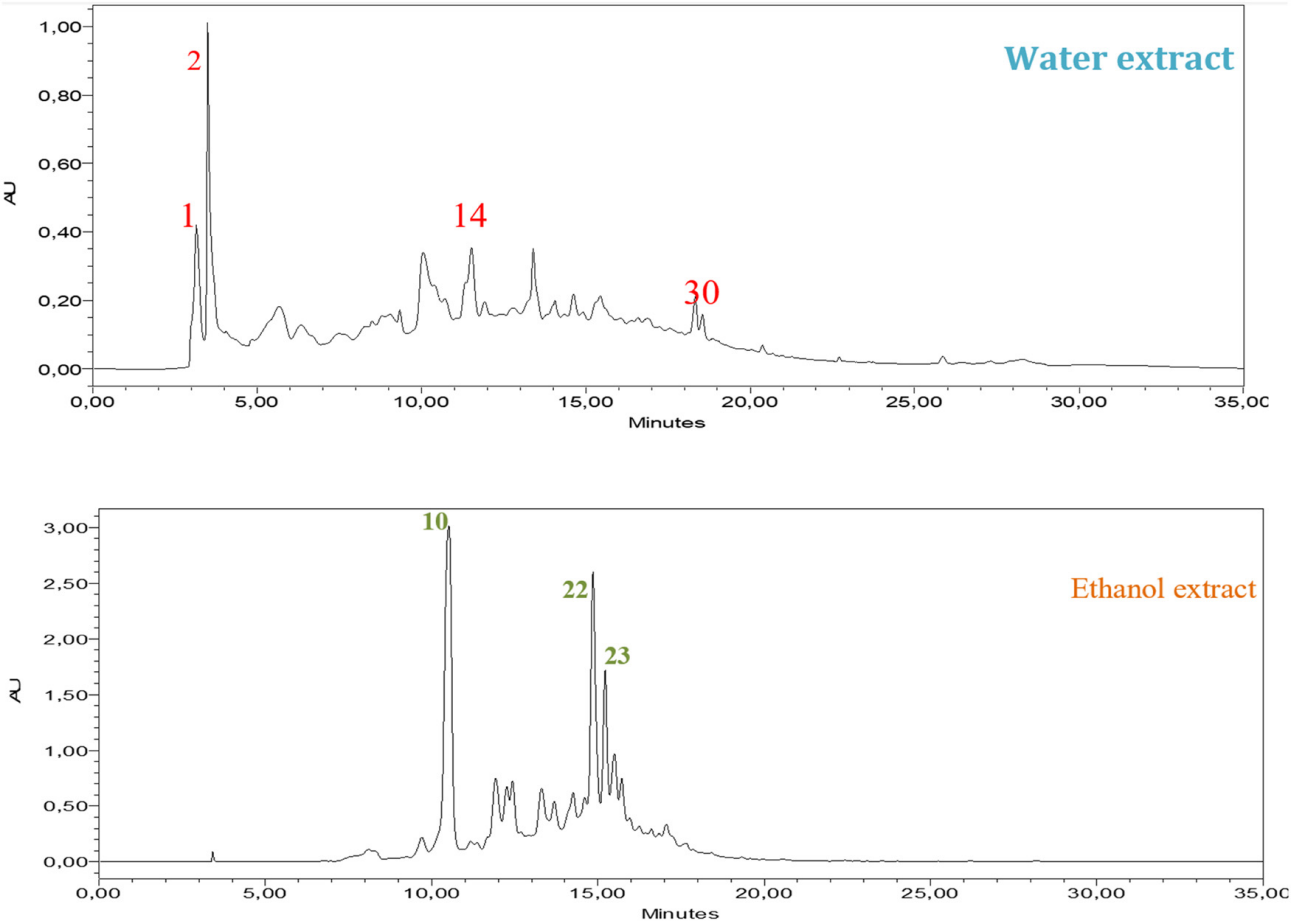
HPLC-DAD chromatogram of extracts from the aerial parts of EF, recorded at 320 nm, in comparison to the standards from the table.

Chromatographic analysis of the aqueous and ethanolic extracts from the aerial sections of the EF plant reveals significant differences in their chemical composition (Table 2, Figure 2). The ethanolic extract is predominantly composed of catechin and gallic acid, which occupy approximately 22.69 and 15.96% of the surface area, respectively. These compounds are widely recognized for their potential health benefits and antioxidant properties. In contrast, the aqueous extract presents a distinct profile, with gallic acid (15.96%) being more abundant than catechin (approximately 32.69%). Several compounds are not detected (ND) in either extract, which could reflect limitations in the analytical method or the extraction process.

**Table 2:** HPLC analysis of compounds detected in extracts from the aerial parts of EF

N	Compounds (%)	Retention time	Ethanol	Water
1	Gallic acid	3.18	ND	15.96
2	Catechin	3.62	ND	22.69
3	Salicylic acid	4.38	ND	3.55
4	NI	5.67	ND	8.03
5	NI	6.34	ND	2.17
6	NI	7.49	ND	1.98
7	NI	8.54	ND	ND
8	Caffeic acid	9.71	1.38	6.48
9	4-Hydroxybenzoic acid	10.03	ND	8.31
10	Catechin hydrate	10.53	24.05	ND
11	Syringic acid	10.93	ND	ND
12	NI	11.15	1.11	ND
13	Vanillic acid	11.36	ND	ND
14	3-Hydroxybenzoic acid	11.84	6.04	10.32
15	NI	12.24	3.47	ND
16	NI	12.42	3.79	ND
17	Vanillin	ND	1.11	ND
18	Naringin	12.98	5.75	ND
19	NI	13.68	3.81	7.84
20	Cinnamic acid	14.06	5.53	1.71
21	Ferulic acid	14.61	3.55	2.16
22	*p*-Coumaric acid	14.84	15.21	ND
23	Sinapic acid	15.24	8.43	ND
24	Succinic acid	15.47	5.65	4.19
25	Quercetin 3-*O*-β-d-glucoside	15.74	3.79	ND
26	Rutin	16.19	1.78	ND
27	NI	16.23	2.23	ND
28	Quercetin	ND	1.15	ND
29	Kaempferol	17.06	2.19	ND
30	Apigénine	18.21	ND	4.87

These results underscore the significant impact of solvent choice on the chemical profiles of plant extracts. The higher concentration of gallic acid in the aqueous extract suggests that water may be more effective in extracting certain phenolic compounds. On the other hand, compounds like catechin hydrate are present exclusively in the ethanolic extract, highlighting the importance of solvent polarity in determining which compounds are extracted from the plant material. The observed differences between the two extraction methods emphasize the need for optimization of extraction conditions to maximize the recovery of bioactive compounds. The chromatographic profiles also demonstrate the occurrence of common bioactive compounds across the different extracts, albeit in varying concentrations depending on the solvent used. These findings are in line with previous studies that reported similar compounds from *Ephedra* extracts [51–53]. Future research could explore further optimization strategies, including varying extraction parameters, to improve the efficacy of isolating specific compounds. A deeper understanding of the extraction process and chemical composition will be crucial for harnessing the pharmacological potential of plant extracts in pharmaceuticals, nutraceuticals, and functional foods.

### 3.2 Electrochemical study

Determining the appropriate duration for electrode immersion in the electrolyte is crucial for achieving electrochemical equilibrium. This study shows that immersing an iron metal plate in 1 M HCl solution for 30 min results in 99% stabilization of the corrosion potential [54–57]. The corrosion potential is significantly influenced by the stability of the working electrode within the electrolyte. As such, it is essential to determine the necessary duration for the corrosion potential to stabilize. The results from this study indicate that 30 min of immersion in 1 M HCl solution is sufficient to achieve nearly complete stabilization, with 99% stabilization of the corrosion potential. This finding is consistent with prior research, highlighting the importance of immersion time in ensuring reliable electrochemical measurements.

#### 3.2.1 OCP monitoring

**Figure 3:**
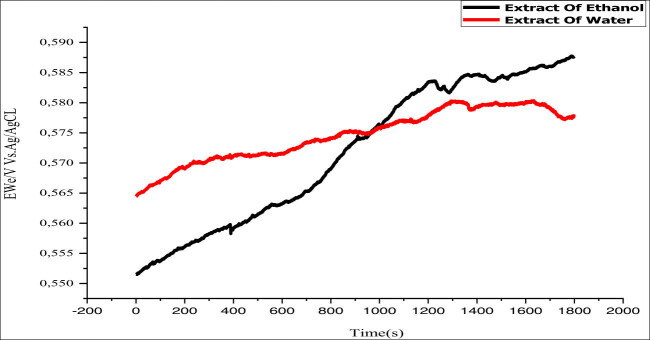
Change in the OCP of MS in a 1 M HCl solution at 298 K, comparing the conditions with and without the addition of 1 g/L of each of the different inhibitor solutions under study.

This method offers a more thorough comprehension of the mechanisms occurring at the metal–electrolyte interface [58,59]. The stability of the OCP was monitored before each polarization and impedance cycles. The OCP variation for the MS electrode in a corrosive environment is shown in Figure 3, with and without 1 g/L EF plant extract at 298 K. Inhibitors cause a slight shift to more negative potentials, indicating their impact on cathodic reactions. Equilibrium potential is reached within 30 min.

#### 3.2.2 Polarization curves (steady-state electrochemical method)

**Figure 4:**
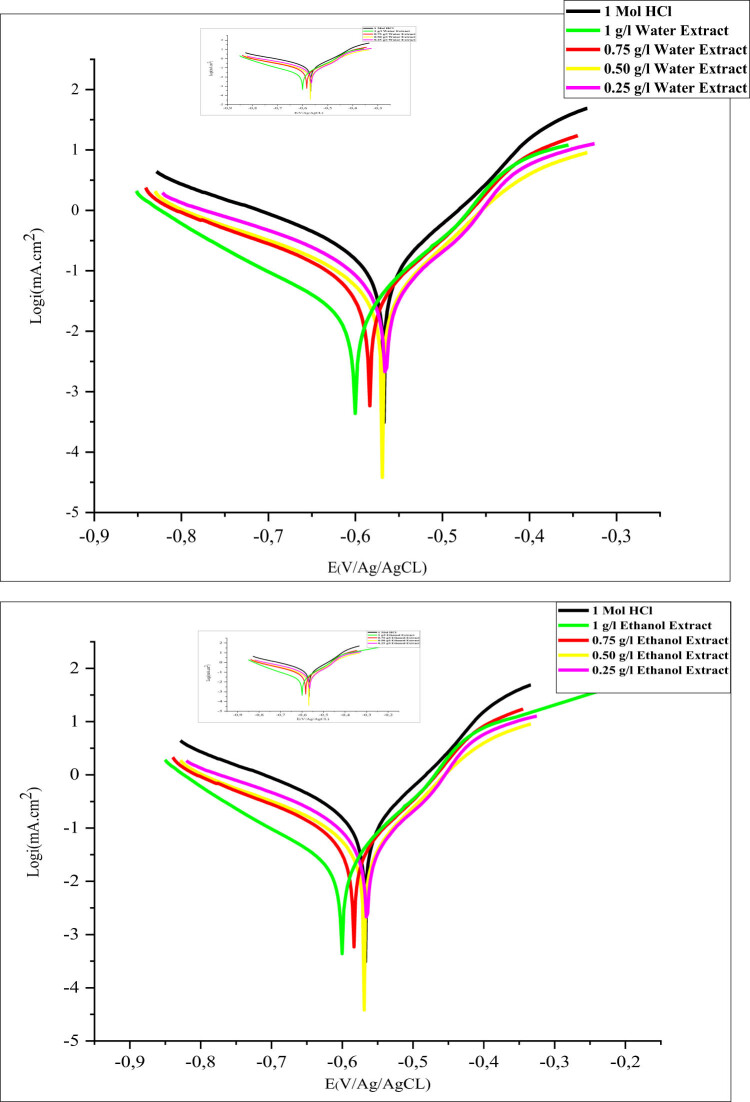
PDP curves for MS in 1 M HCl with and without different EF concentrations at 298 K.

This study employed stationary electrochemical techniques to evaluate the effectiveness of EF plant extracts sourced from both water and ethanol. The primary methods involve analyzing current/voltage curves under potentiodynamic conditions, considering both anodic and cathodic potentials. Electrochemical metrics, including Tafel slopes (*ß*_c_, *ß*_a_), corrosion potential, and corrosion current densities, are precisely noted in Table 3. Figure 4 illustrates the polarization curves of MS in an acidic medium, with and without various inhibitors. The efficiency of corrosion inhibition (IE%) was computed using equation (7).

(7) \[\text{IE} \% =\frac{{i}_{\text{corr}/B}-{i}_{\text{corr}/\text{inh}}}{{i}_{\text{corr}/B}}\times 100,]\]

where *i*_corr/*B*_ represents the current density of the blank solution, which contains only HCl, and *i*_corr/inh_ represents the current density in the presence of inhibitor (medium containing the studied inhibitor).

**Table 3:** Electrochemical parameters derived from potentiodynamic polarization (PDP) curves for MS corrosion in a 1 M HCl solution at 298 K, with varying concentrations of EF extract

	Conc.	−*E*_corr_	*i*_corr_	*i*_corr_	−*β*_c_	*η*_PDP_
	(g/l)	mV/Ag/Agcl	µA/cm^2^	mV/dec^1^	mV/dec^1^	%
1 M HCl	—	498	983	130	128	—
Ethanol	1	406	31	127	82	**96.84**
	0.75	408	41	126	109	95.82
	0.5	410	49	124	112	95.01
	0.25	403	62	130	114	93.69
Water	1	404	38	126	86	**96.13**
	0.75	401	52	130	89	**94.71**
	0.50	402	60	132	114	**93.89**
	0.25	409	67	130	115	93.18

Bold values correspond to the concentrations that allowed us to achieve greater inhibition of corrosion.

The stability observed in all samples can be attributed to the formation of an iron oxide/hydroxide layer on the MS surface due to the presence of inhibitor. Polarization curves (Figure 4) illustrate both cathodic and anodic reactions in a 1 M HCl solution with MS under conditions with and without the tested inhibitors, at concentrations ranging from 0.25 to 1 g/L at 25°C. Data from Table 3 reveal a gradual decrease in the corrosion current density (*i*_corr_) as the concentration of EF inhibitors increases. For instance, with the ethanolic extract, the current density is 62 µA/cm² at a concentration of 0.25 g/L, dropping significantly to approximately 31 µA/cm² at 1 g/L. Tafel analysis shows that the inhibition efficiency increases with increasing inhibitor concentration, reaching 96.13% with the aqueous extract and 96.84% with the ethanolic extract, both at a concentration of 1 g/L. Electrochemical parameters, including the cathodic Tafel slope (*β*_c_), corrosion potential (*E*_corr_), inhibition efficiency (*η*_PDP_), and corrosion current density (*i*_corr_), were determined and are summarized in Table 2. The shifts in corrosion potential (*E*_corr_) remained within ±85 mV, confirming that EF mitigates the corrosive effects of HCl through a mixed-type inhibition mechanism, as demonstrated by the variation in *E*_corr_ within the ±85 mV range [60–62]. These results indicate that the presence of inhibitor alters both anodic and cathodic current rates. The consistent cathodic curves across all samples suggest that the hydrogen reduction mechanism is governed solely by activation kinetics. The EF extract also affects the iron dissolution process in the acidic environment, promoting the formation of a protective layer. This aligns with the observed variations in corrosion potential (*E*_corr_), which remains within the ±85 mV range, signifying a mixed-type inhibition mechanism that effectively reduces HCl’s corrosive impact [61,63–65].

#### 3.2.3 Electrochemical impedance spectroscopy (EIS)

**Figure 5:**
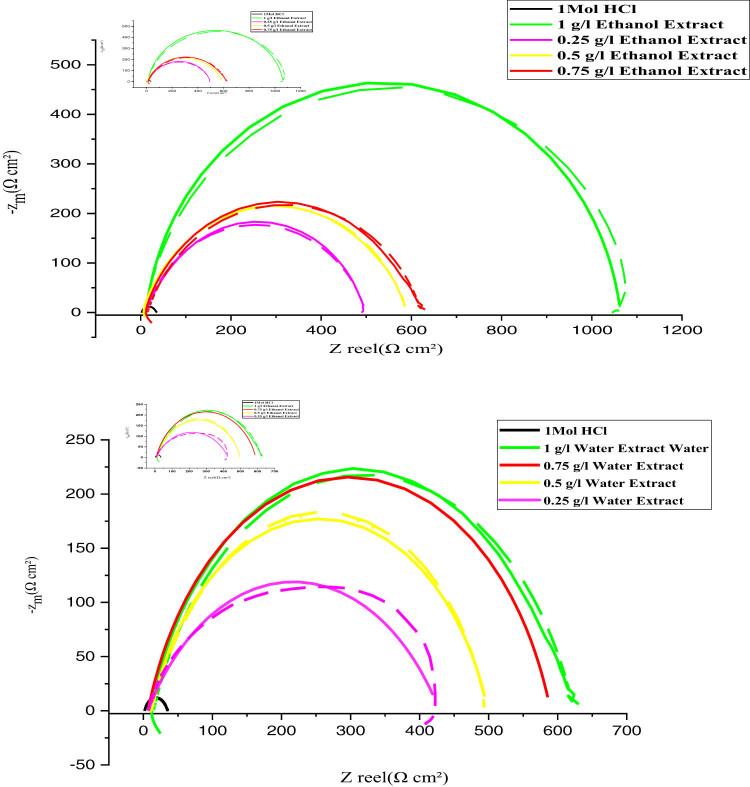
Nyquist diagrams for inhibitors extracted from the aerial part of the EF plant at 298 K at different concentrations.

**Figure 6:**
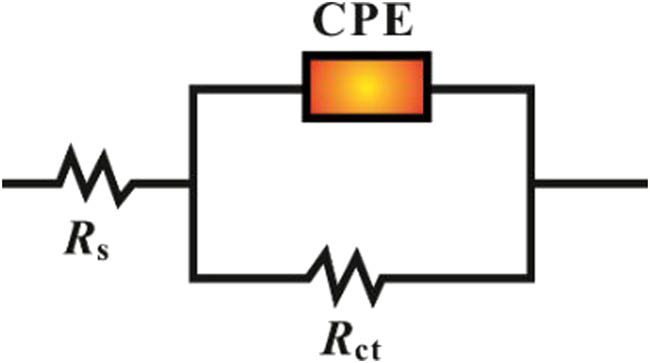
Equivalent circuit model for electrochemical data fitting.

EIS analysis provided significant insights into the electrochemical behavior of MS in a 1 M HCl solution with and without the addition of EF extracts. In the absence of an inhibitor, the impedance spectrum displayed a minor semicircle, reflecting rapid iron degradation and hydrogen evolution. Conversely, when EF extracts were added, a high-frequency capacitive loop appeared, and the semicircle enlarged with increasing inhibitor concentrations (Figure 5). The frequency dispersion observed in the impedance spectrum can be attributed to surface irregularities [66], the presence of contaminants or imperfections [56], active center dispersion, and the formation of porous protective layers [63]. Incorporating a constant phase element (CPE) accounted for the observed frequency dispersion (Figure 6). The CPE parameter was calculated using equation 8 [67]:

(8) \[{Z}_{{\mathrm{cpe}}}\left={Q}^{-1}({i\omega )}^{-n},]\]

where *Q* denotes the amplitude of the CPE, *i* signifies the imaginary component of the CPE, *ω* represents the rotational frequency, and *n* denotes the experimental coefficient.

(9) \[\text{IE} \% =\frac{{R}_{\text{ct}/\text{inh}}-{R}_{\text{ct}/\text{B}}^{\text{'}}}{{R}_{\text{ct/B}}}\times 100.]\]

Table 4 presents the electrochemical parameters. Solution resistance (*R*_s_) values ranged from 1.03 to 2.08 Ω/cm², indicating high conductivity in the solution. The charge transfer resistance (*R*_ct_) increased significantly upon the addition of inhibitors, from 22.2 Ω/cm² for the blank sample to a range of 425.1 to 1,075 Ω/cm² [8], confirming the inhibitor’s effectiveness in mitigating corrosion. Additionally, double-layer capacitance (*C*_dl_) decreased from 89.10 µF/cm² in the absence of inhibitors to a range of 24.43 to 50.78 µF/cm² with inhibitors, suggesting slower charge–discharge rates at the metal–electrolyte interface. The phase shift values (*n*_dl_) approached unity, ranging between 0.8 and 0.83, indicating that the electrical double layer created by the adsorption of bioactive molecules from the EF extract acted as a pseudo-capacitor.

**Table 4:** EIS values for MS in corrosive media (1 M HCl) without and with inhibitors (EF extracts)

	Conc.	*R*_s_	*R*_ct_	*C*_dl_	*n*_dl_	*Q* (µF/sn)	*Ɵ*	*χ*²	*ƞ*_imp_
	(g/L)	(Ω/cm^2^)	(Ω/cm^2^)	(µF/cm^2^)					%
1 M HCl	—	1.70	22.2	89.10	0.784	312.7	—	0.003	—
Ethanol	1	1.25	1075.0	43.05	0.837	80.70	0.930	0.002	97.93
	0.75	1.20	620.5	44.71	0.829	85.32	0.926	0.004	96.40
	0.50	1.04	596.5	45.50	0.827	90.23	0.923	0.005	96.2
	0.25	1.60	500.7	50.78	0.823	110.56	0.864	0.003	95.54
Water	1	1.50	630.7	24.43	0.816	59.70	0.944	0.003	96.48
	0.75	2.08	597.0	31.44	0.817	62.66	0.922	0.005	96.28
	0.5	1.74	498.0	42.30	0.805	94.93	0.909	0.005	95.54
	0.25	1.03	425.1	50.56	0.825	134.70	0.856	0.004	94.77

The corrosion inhibition efficiency was significantly enhanced by the addition of EF extracts, achieving a maximum value of 97.93% at higher concentrations. The results indicate that EF extracts effectively inhibit the corrosion of MS in acidic environments through multiple mechanisms. The enlargement of the semicircle in the impedance spectrum and the presence of a high-frequency capacitive loop demonstrate that the inhibitor influences both the charge transfer process and the adsorption of inhibitory molecules. The frequency dispersion observed at the metal–electrolyte interface can be linked to surface irregularities, imperfections, and the formation of an organic protective film on the steel surface [56,63,66]. The significant increase in charge transfer resistance (*R*_ct_) and the reduction in double-layer capacitance (*C*_dl_) further support the hypothesis that EF extracts form a robust organic layer, limiting the active sites for corrosion reactions. The incorporation of a CPE to model the system acknowledges the heterogeneous nature of the interface, as conventional circuit models proved insufficient [67]. The observed reduction in corrosion processes, as evidenced by increased *R*_ct_ values and decreased *C*_dl_, highlights the effectiveness of EF extracts. These findings confirm the ability of EF extracts to act as efficient, eco-friendly corrosion inhibitors, with the inhibition efficiency reaching a maximum of 97.93% [8]. Such natural inhibitors are valuable alternatives for mitigating corrosion in industrial applications, especially in acidic environments.

### 3.3 Surface morphological studies

**Figure 7:**
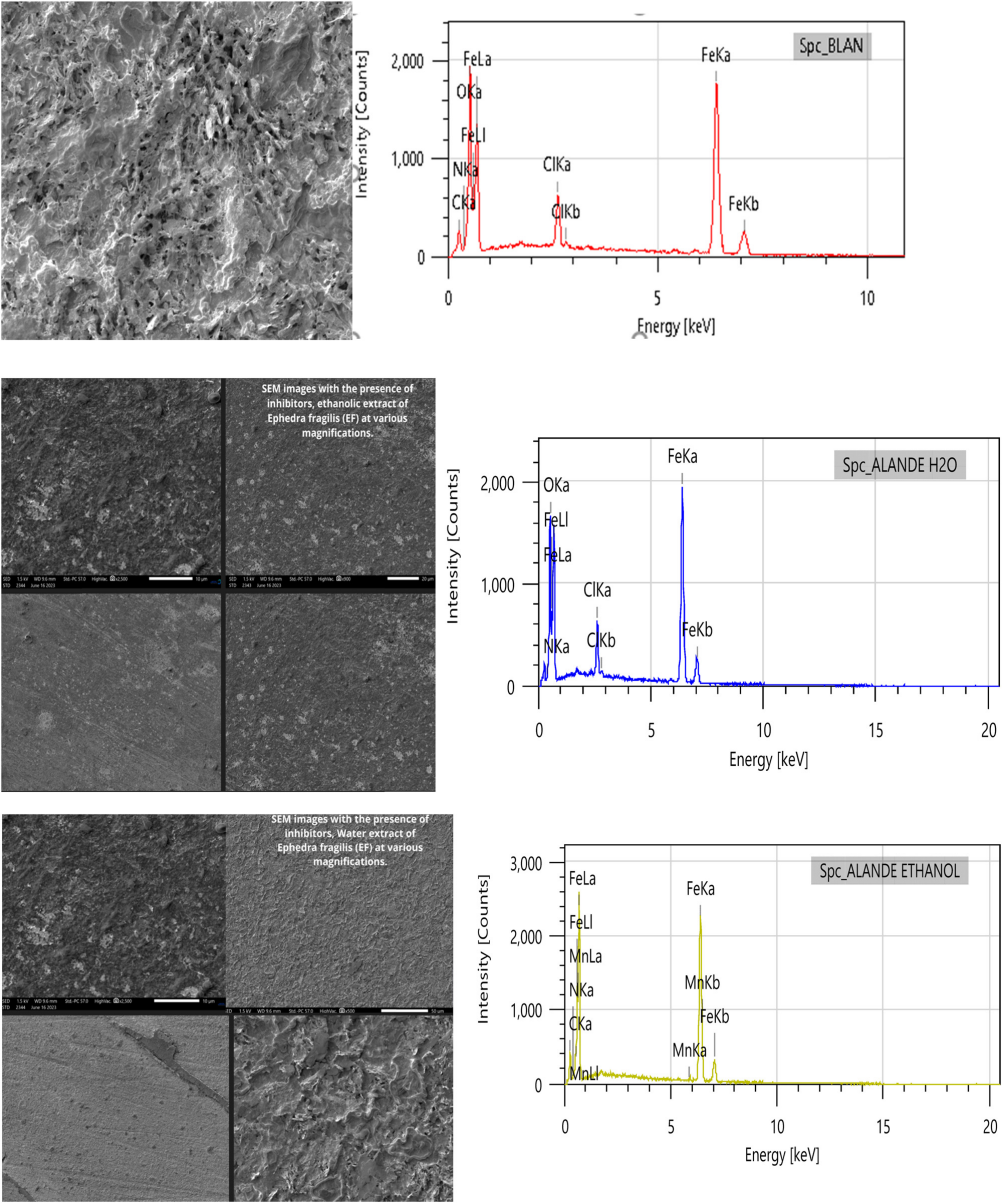
SEM–EDX images of MS samples immersed for 6 h in 1 M HCl solution, both with and without aqueous EF extract inhibitors.

Figure 7 shows the surface characteristics of MS samples after 6 hof immersion in 1 M HCl, both with and without the addition of 1 g/L various EF extracts. As shown in Figure 7(a), the untreated steel surface is visibly corroded, showing irregularities caused by the corrosive effects of the acid. This suggests that in the absence of an inhibitor, the steel undergoes significant degradation due to the aggressive action of the HCl. In contrast, Figure 7(b) and (c) displays the surfaces of samples treated with 1 g/L ethanolic and aqueous EF extracts, respectively. These surfaces appear much smoother and more uniform compared to the untreated steel. This improvement in surface morphology indicates that the inhibitors are effective at adsorbing onto the metal surface, forming a protective layer. This protective layer helps to mitigate the corrosion process, preserving the integrity of the metal and preventing further damage from the corrosive environment [68,69].

This observation suggests that EF extracts act as effective corrosion inhibitors by forming a protective film on the steel surface, which prevents direct contact between the metal and the acidic solution, thereby reducing the overall corrosion rate.

Table 5 presents the weight percentages of various elements on the steel surface, determined by EDX analysis in a 1 M HCl solution, conducted at 298 K with and without inhibitors at a concentration of 1 g/L. The EDX spectrum of the MS sample without inhibitors reveals distinct peaks for major elements, including silicon (Si), aluminum (Al), carbon (C), manganese (Mn), chromium (Cr), and iron (Fe). In the absence of inhibitors, the mass percentages show significant amounts of chloride (Cl) and oxygen (O), with Cl at 3.96% and O at 13.75%. These high levels of chlorine and oxygen indicate the formation of corrosion products on the steel surface, highlighting the aggressive nature of the acid. When the MS sample was immersed in the solution with EF inhibitors, there is a noticeable reduction in the levels of chloride and oxygen. Specifically, the ethanolic extract completely eliminates the presence of chloride and oxygen, while the aqueous extract shows a significant reduction in these elements. This decrease in chlorine and oxygen concentrations reflects the protective action of the inhibitors, which help reduce chloride ion infiltration and prevent iron oxidation. This suggests that the inhibitors form a protective film on the steel surface, mitigating corrosion and preserving the integrity of the metal. These findings support the idea that EF extracts act effectively as corrosion inhibitors by forming a protective layer that shields the metal from the corrosive effects of HCl.

**Table 5:** Mass percentages of different components on the steel surface measured using EDX

Element	wt% MS with HCl	% By weight MS with ethanol inhibitors	% By weight MS with water inhibitors
C	4.74 ± 0.06	6.75 ± 0.06	—
N	—	0.08 ± 0.04	—
O	13.75 ± 0.11	—	10.77 ± 0.10
Cl	3.96 ± 0.06	—	1.14 ± 0.07
Fe	77.55 ± 0.51	92.15 ± 0.53	88.08 ± 0.54
Mn	—	1.02 ± 0.08	—
Si	—	—	—

This analysis was conducted in a 1 M HCl solution at 298 K, both with and without inhibitors at a concentration of 1 g/L.

### 3.4 Theoretical method results

**Figure 8:**
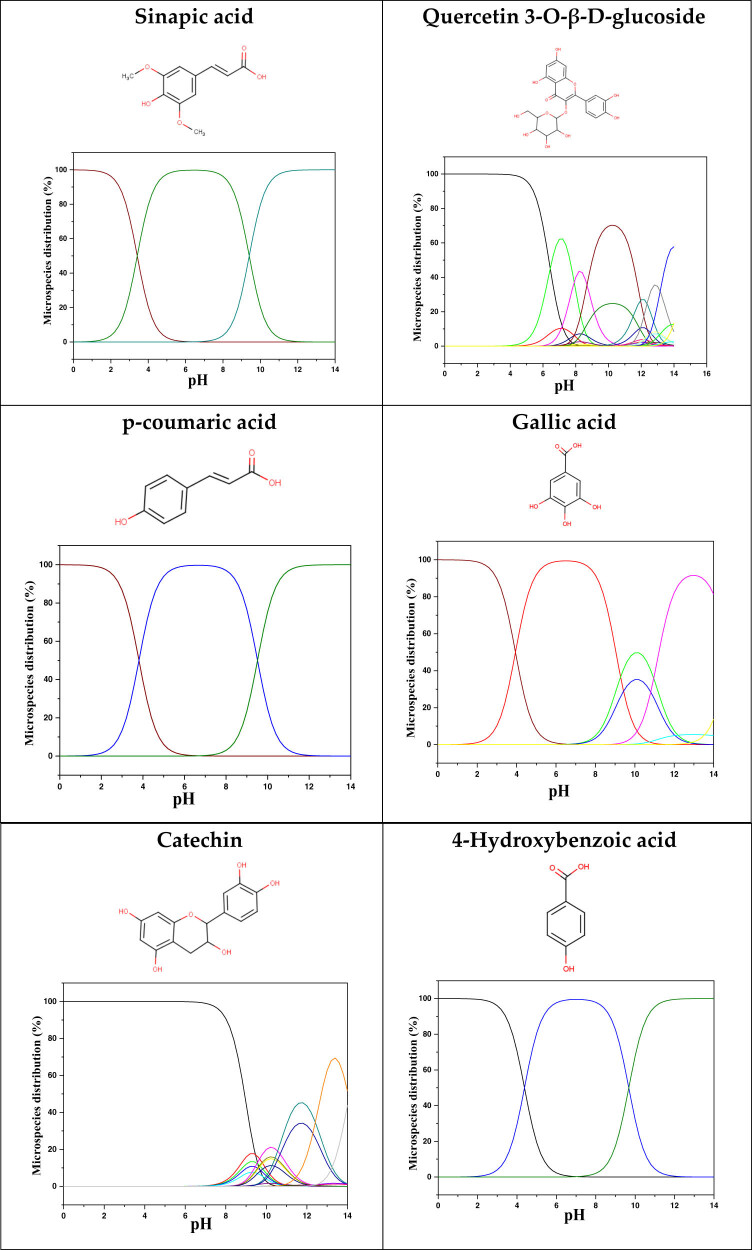

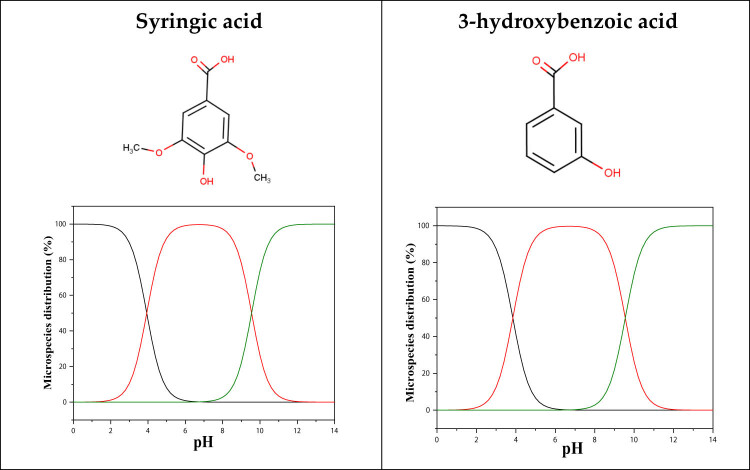
Microspecies distribution versus pH.

Quantum calculations are crucial for understanding and classifying the activity of molecules utilized as corrosion inhibitors and their interactions with the MS surface. Consequently, prior to these calculations, the anticipated protonated forms of the molecules under study were analyzed using Marvinsketch software [31,70]. Figure 8 illustrates the species distribution as a function of pH. Theoretical computations were performed for the neutral state in the aqueous phase, with the resulting descriptors detailed in Table 7. The protonation results reveal that all molecules predominantly exist in their neutral form within the acidic range. Therefore, it can be concluded that these neutral forms are the expected configurations likely to be present in the 1 M HCl solution under study.

**Table 7:** Optimized molecular structures, LUMO and HOMO distributions, and electrostatic potential maps for the studied compounds

	Optimized molecules	HOMO	LUMO	ESP map
Sinapic acid	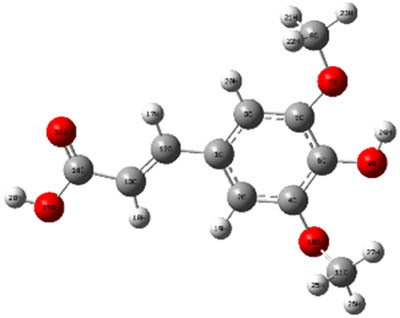	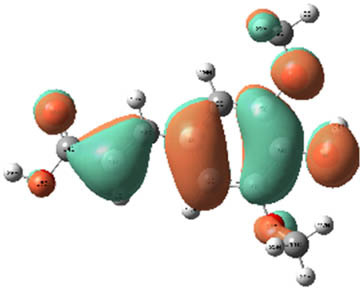	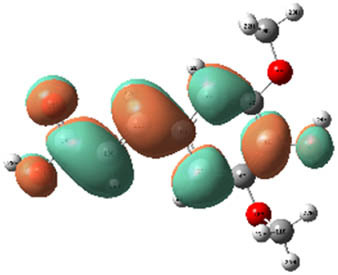	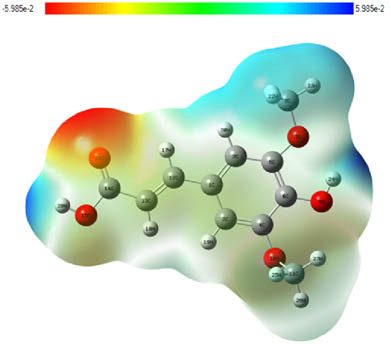
*p*-Coumaric acid	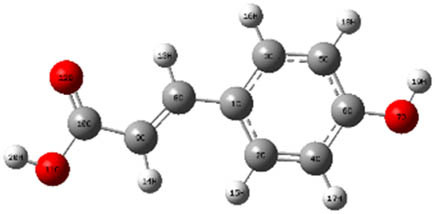	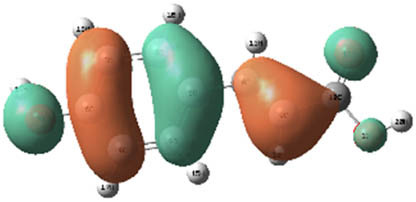	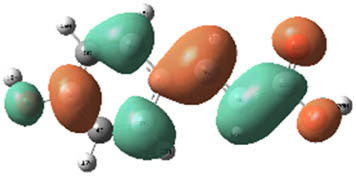	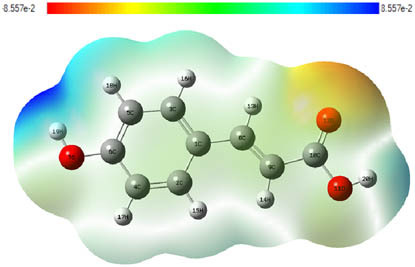
Catechin	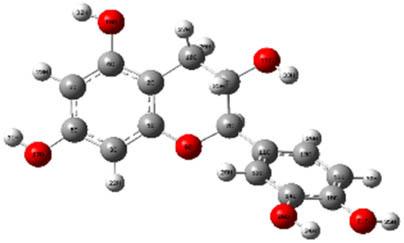	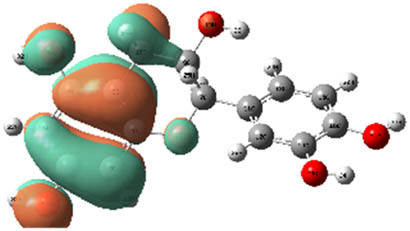	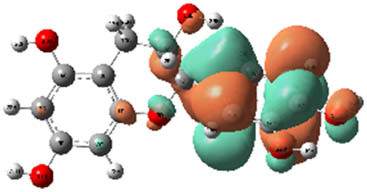	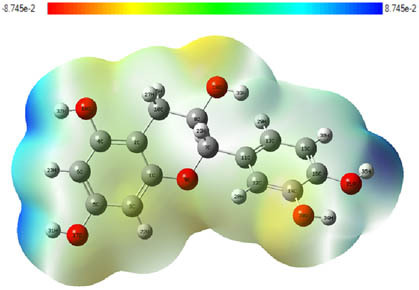
3-Hydroxybenzoic acid	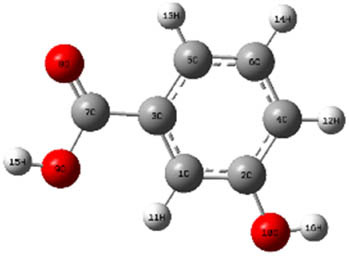	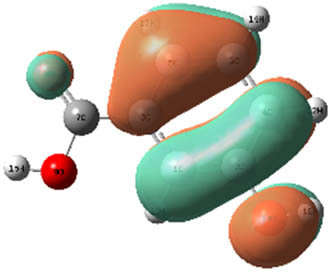	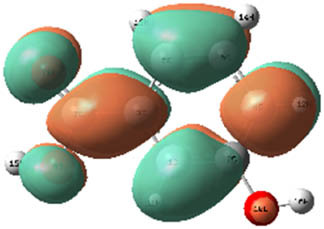	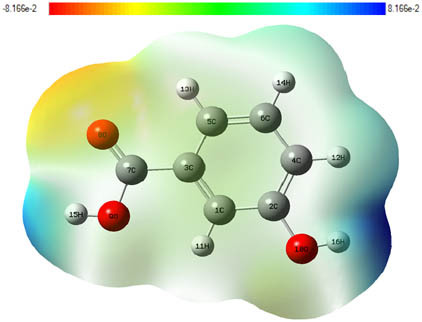
Gallic acid	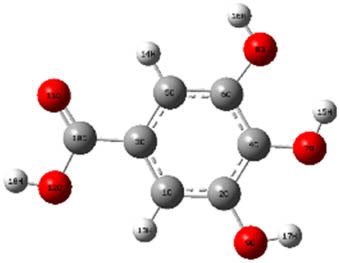	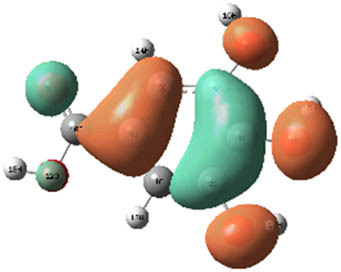	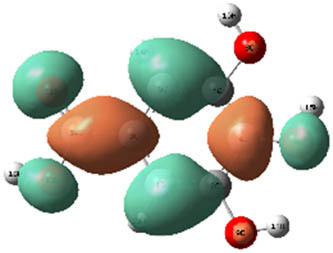	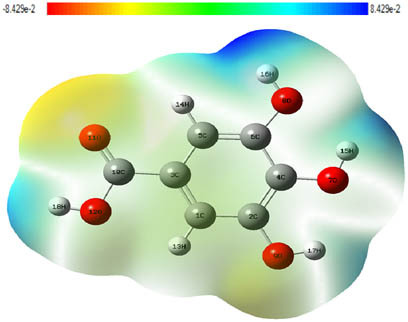

Table 6 provides a detailed analysis of the key characteristics of the neutral forms in the primary structure of EF extracts. The parameters studied include the levels of the highest occupied molecular orbital (HOMO), the lowest unoccupied molecular orbital (LUMO), dipole moment values, and the Δ*E*_gap_. These indicators are crucial for evaluating the anti-corrosive potential of the extracted compounds, as referenced in the study of Rehman et al. [71,72].

**Table 6:** Reactivity descriptors for neutral molecules in the aqueous phase

	Descriptors	*E*_HOMO_ (eV)	*E*_LUMO_ (eV)	Δ*E*_gap_ (eV)	*Ƞ* (eV)	*σ* (eV^−1^)	*χ* (eV)	Δ*E*_back-donation_	Δ*N*_Fe/110_
Ethanol	Sinapic acid	−5.9855	−2.0335	3.9519	1.9759	0.5060	4.0095	−0.4939	0.2050
	*p*-Coumaric acid	−6.2266	−1.9965	4.2301	2.1150	0.4728	4.1115	−0.5287	0.1674
	Catechin	−6.0320	−0.4378	5.5942	2.7971	0.3575	3.2349	−0.6992	0.2833
Water	Catechin	−6.0320	−0.4378	5.5942	2.7971	0.3575	3.2349	−0.6992	0.2833
	3-Hydroxybenzoic acid	−6.5861	−1.6572	4.9289	2.4644	0.4057	4.1216	−0.6161	0.1416
	Gallic acid	−6.2824	−1.4305	4.8518	2.4259	0.4122	3.8564	−0.6064	0.1985

The results highlight particularly low Δ*E*_gap_ values for several compounds in the extracts. For the ethanolic extract, the lowest values were recorded for sinapic acid (3.9519 eV), *p*-coumaric acid (4.2301 eV), and catechin (5.5942 eV). In comparison, for the aqueous extract, the lowest Δ*E*_gap_ values were observed for gallic acid (4.8518 eV), 3-hydroxybenzoic acid (4.9289 eV), and catechin (5.5942 eV). These findings suggest that compounds with lower Δ*E*_gap_ values are more chemically reactive, enhancing their ability to interact with metallic surfaces and thereby exhibiting significant anti-corrosive activity. The presence of such compounds in both extract types demonstrates their potential as corrosion inhibitors.

The number of electrons transferred (ΔNFe/110) provides insight into the ability of these molecules to act as electron donors. The positive values confirm this capability, which is critical for improving interactions with metallic surfaces, especially in corrosive environments. Moreover, the molecular hardness (*η*) values indicate that the stability of the extracted molecules, regardless of their origin (ethanolic or aqueous), is relatively consistent. This molecular stability contributes to the durability and effectiveness of the compounds as corrosion inhibitors.

In conclusion, the data presented in Table 6 clearly illustrate the role of the active compounds in EF extracts in preventing corrosion. The Δ*E*_gap_ values, combined with the electronic properties and molecular stability, confirm their potential as natural and effective solutions for corrosion prevention.

DFT is essential for assessing the inhibitory effectiveness of molecules from the extract when they adsorb onto MS surfaces. This adsorption process depends on donor–acceptor interactions shaped by the molecules’ electronic structure. Electron-dense areas of these molecules can transfer their charges to the metal–liquid interface, particularly interacting with the unoccupied orbitals of iron atoms.

Table 7 displays the electronic structure of molecules derived from the aerial parts of the EF plant, outlining the distribution of the HOMO and LUMO. The data indicate that the HOMO is predominantly situated on the benzene ring, methoxy group, and alcohol group in compounds such as sinapic acid, *p*-coumaric acid, gallic acid, 3-hydroxybenzoic acid, and catechin. Conversely, the HOMO extends across the entire molecule's surface, reflecting the presence of π electrons from the benzene rings and lone electron pairs from heteroatoms. This arrangement makes these electrons available for interaction with metal orbitals at the interface. Meanwhile, the LUMO is mainly found on the benzene ring and the oxygen/carbon atoms. Consequently, molecules from the EF extract are capable of adsorbing onto the iron surface by accepting electrons from the iron’s filled orbitals.

**Figure 9:**
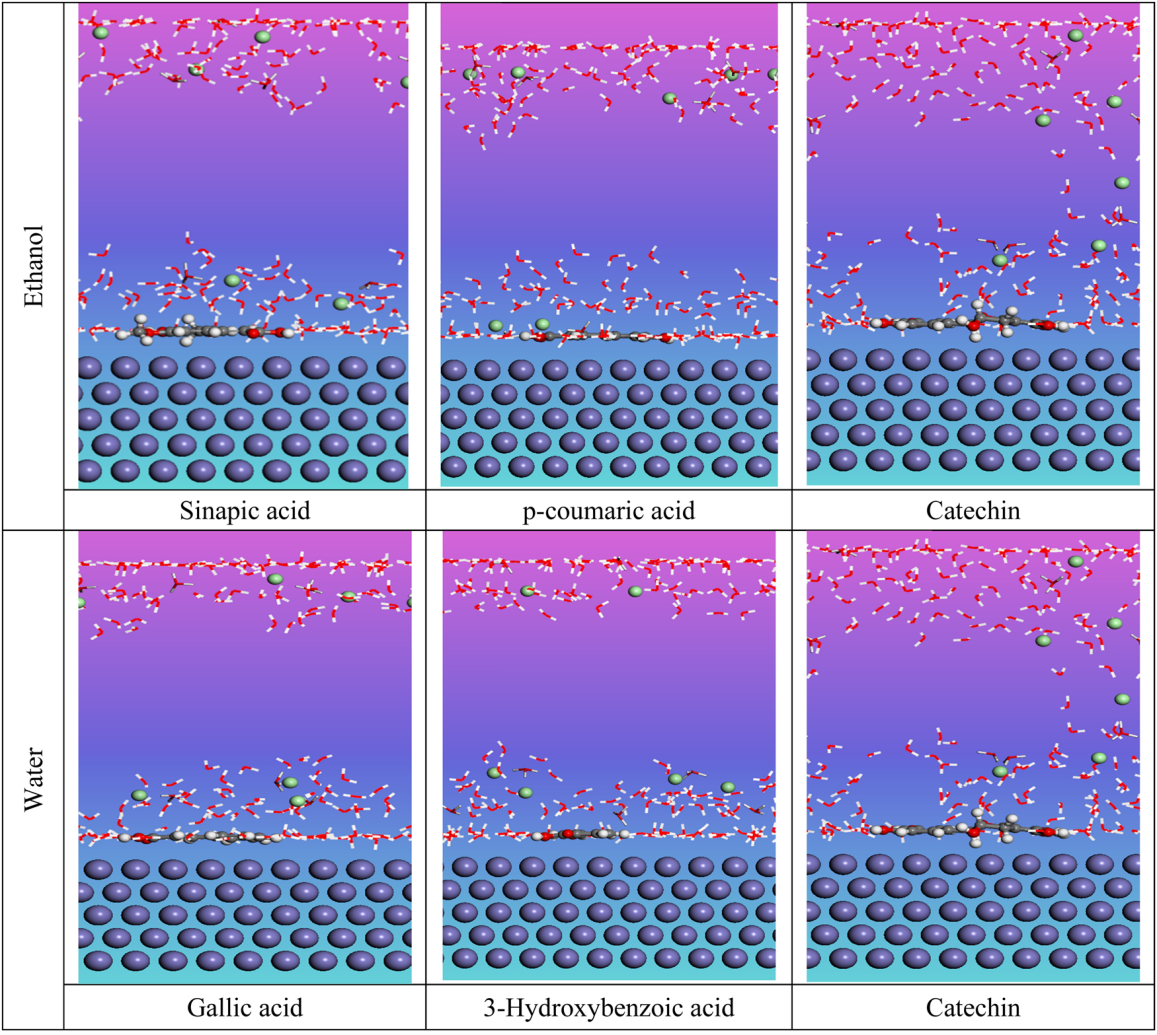
Side views of the most stable configuration for the studied molecules.

Additionally, Monte Carlo (MC) simulations were conducted on Fe 110/200 H_2_O systems to deepen our understanding of how molecular structures interact with the iron surface. The most stable adsorption configurations of the inhibitors are shown in Figure 9, featuring both top and cross-sectional views.

In our research on the effectiveness of various chemical structures as corrosion inhibitors, we observed that sinapic acid exhibits greater reactivity than *p*-coumaric acid and catechin in the ethanolic extract. On the other hand, catechin shows substantial reactivity compared to other compounds in the aqueous extract (Table 8). Simulation studies involving the Fe (110) surface and 200 water molecules show that the identified inhibitors adhere to the iron surface, with differences in their adsorption behavior.

**Table 8:** MC simulation descriptors

	Descriptors	*E*_ads_	Inhibitor: d*E*_ad_/d*N*_*i*_	H_2_O: d*E*_ad_/d*N*_*i*_	H_3_O^+^: d*E*_ad_/d*N*_*i*_	Cl^−^: d*E*_ad_/d*N*_*i*_
Ethanol	Sinapic acid	−3855.70	−158.51	−12.86	−147.40	−143.63
	*p*-Coumaric acid	−3759.53	−119.11	−10.38	−147.85	−152.96
	Catechin	−3490.41	−192.30	−12.91	−148.52	−124.30
Water	Catechin	−3490.41	−192.30	−12.91	−148.52	−124.30
	3-Hydroxybenzoic acid	−3709.56	−102.36	−12.14	−149.03	−149.90
	Gallic acid	−3713.40	−118.20	−8.05	−138.61	−146.11

To summarize the findings, catechin in the ethanolic extract binds to fewer active sites on the iron surface compared to sinapic acid. In contrast, in the aqueous extract of EF, gallic acid is absorbed with fewer reactive sites than catechin, which can be attributed to the non-polar nature of alkyl chains present in the molecular structure. These observations are supported by the adsorption energy data, where the order of adsorption energy in the EF ethanolic extract is as follows: sinapic acid > *p*-coumaric acid > catechin. In the EF aqueous extract, the order is catechin > 3-hydroxybenzoic acid > gallic acid. These results correlate with the DFT calculations provided in Table 7.

Additionally, it is noteworthy that the adsorption energy of water is considerably lower than that of the other molecules tested, suggesting that water molecules tend to migrate away from the iron surface, further supporting the preferential adsorption of the inhibitor molecules.

### 3.5 Inhibition mechanism

**Figure 10:**
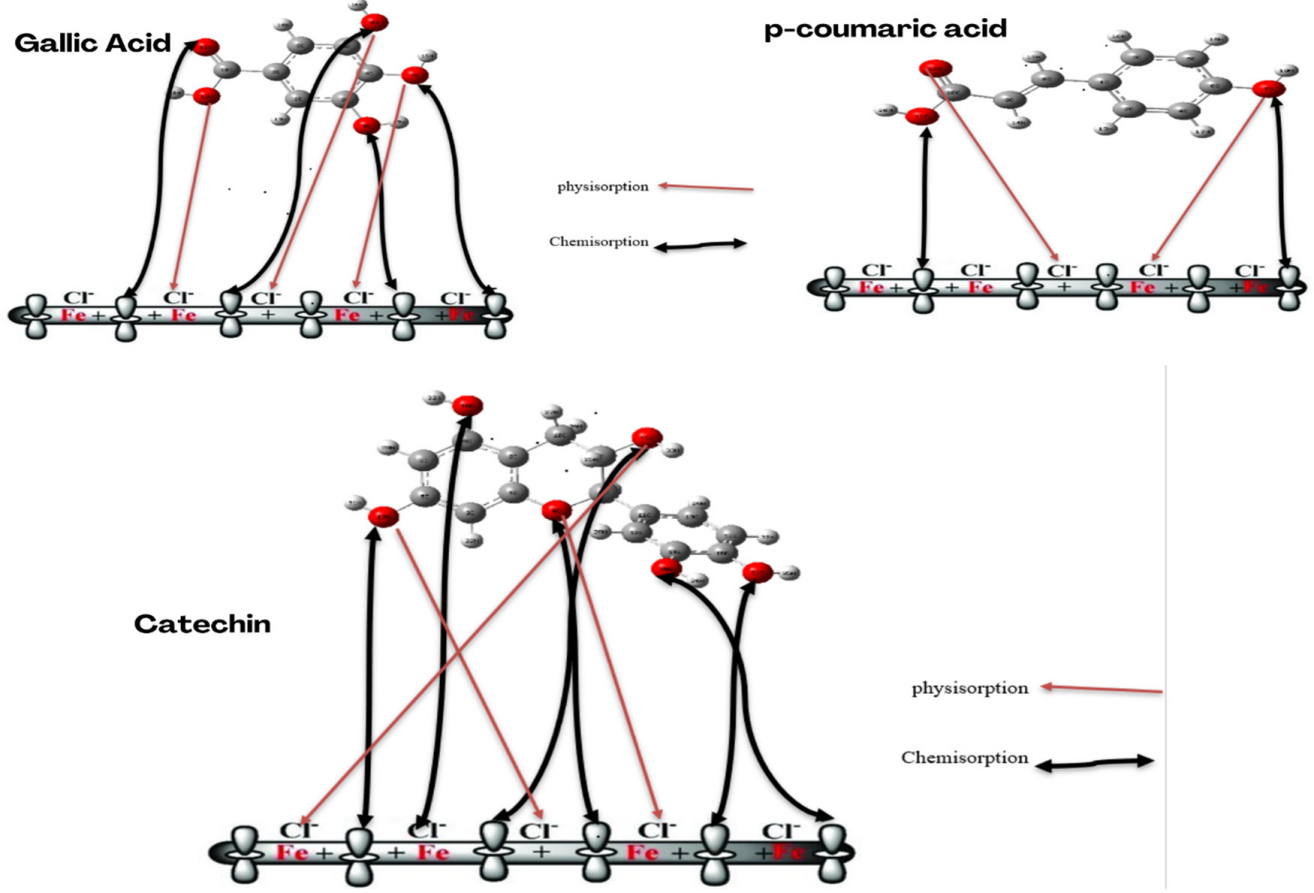
Adsorption model of macromolecules on the MS surface.

The adsorption of inhibitory molecules extracted from the aerial part of EF can occur on the surface of MS through physical, chemical, or a combination of both mechanisms. In the absence of an inhibitor, negatively charged chloride ions are observed to be adsorbed onto the surface of untreated steel, resulting in damage. We have described the mechanism by which the main organic molecules extracted from EF form a film at the MS–solution interface, effectively protecting steel against corrosion. The principal molecules found in both extracts studied are gallic acid, catechin, and *p*-coumaric acid (Figure 10). We have observed that these molecules can all transfer π electrons to iron cations containing empty 3D orbitals, leading to chemisorption. In our case, the π electrons from oxygen atoms can interact with iron cations. Charged inhibitory molecules involve an electrostatic interaction with metal ions, corresponding to physisorption. Our study revealed the existence of electrostatic forces between the ionic charge of the inhibitors and the electrically charged metal surface. The figure illustrates the mechanism of corrosion inhibition by the three principal molecules extracted from the aerial part of EF [73–75].

## 4 Conclusions

In conclusion, our research demonstrates the significant potential of EF plant extracts, particularly the aqueous and ethanolic extracts, as effective corrosion inhibitors for MS. Both extracts exhibited impressive inhibition efficiencies, with values of 96.26 and 95.4%, respectively. This corrosion protection can be attributed to the formation of a protective layer on the steel surface, which significantly reduces its dissolution in an acidic environment. Through HPLC, key inhibitory compounds, such as catechin and gallic acid, were identified, suggesting that these bioactive molecules play a central role in corrosion inhibition. Further investigations into the mechanisms of inhibition revealed that these molecules adsorb onto the steel surface, effectively preventing corrosion, especially under aggressive acidic conditions. Additionally, simulation techniques were employed to gain a deeper understanding of the underlying corrosion inhibition mechanisms, offering valuable insights into how these inhibitor molecules interact with the metal surface. Overall, the results highlight the potential of EF extracts as eco-friendly and efficient corrosion inhibitors, offering a viable alternative to traditional synthetic inhibitors.
